# Characterization of diverse natural variants of CYP102A1 found within a species of *Bacillus megaterium*

**DOI:** 10.1186/2191-0855-1-1

**Published:** 2011-03-28

**Authors:** Ji-Yeon Kang, So-Young Kim, Dooil Kim, Dong-Hyun Kim, Sun-Mi Shin, Sun-Ha Park, Keon-Hee Kim, Heung-Chae Jung, Jae-Gu Pan, Young Hee Joung, Youn-Tae Chi, Ho Zoon Chae, Taeho Ahn, Chul-Ho Yun

**Affiliations:** 1 School of Biological Sciences and Technology, Chonnam National University, Gwangju 500-757, Republic of Korea; 2 Systems Microbiology Research Center, Korea Research Institute of Bioscience and Biotechnology, Daejeon 305-806, Republic of Korea; 3 Department of Biochemistry, College of Veterinary Medicine, Chonnam National University, Gwangju 500-757, Republic of Korea

## Abstract

An extreme diversity of substrates and catalytic reactions of cytochrome P450 (P450) enzymes is considered to be the consequence of evolutionary adaptation driven by different metabolic or environmental demands. Here we report the presence of numerous natural variants of P450 BM3 (CYP102A1) within a species of *Bacillus megaterium*. Extensive amino acid substitutions (up to 5% of the total 1049 amino acid residues) were identified from the variants. Phylogenetic analyses suggest that this P450 gene evolve more rapidly than the rRNA gene locus. It was found that key catalytic residues in the substrate channel and active site are retained. Although there were no apparent variations in hydroxylation activity towards myristic acid (C_14_) and palmitic acid (C_16_), the hydroxylation rates of lauric acid (C_12_) by the variants varied in the range of >25-fold. Interestingly, catalytic activities of the variants are promiscuous towards non-natural substrates including human P450 substrates. It can be suggested that CYP102A1 variants can acquire new catalytic activities through site-specific mutations distal to the active site.

## Introduction

Cytochrome P450s (EC 1.14.14.1; P450 or CYP) are remarkably diverse oxygenation catalysts that are found throughout all classes of life. Although over 11,200 genes of P450s have been found in archaea, bacteria, fungi, plants, and animals (the Cytochrome P450 homepage, http://drnelson.uthsc.edu/P450.statsfile.html), their evolution is not clear. An extreme diversity of substrates and catalytic reactions is characteristic of P450s (Guengerich 2001) and is considered to be the consequence of evolutionary adaptation driven by different metabolic or environmental demands in different organisms. Although most bacterial P450s do not seem to be essential to basic metabolism, they have important roles in the production of secondary metabolites and in detoxication (Kelly et al. 2005).

P450 BM3 (CYP102A1) from *Bacillus megaterium *is a self-sufficient monooxygenase as it is fused to its redox partner, an eukaryotic-like diflavin reductase. Interestingly, sequence analysis for the P450 phylogenetic tree suggested that the CYP102A1 clusters with the eukaryotic P450s but not with other prokaryotic P450s (Lewis et al. 1998). The natural substrates of CYP102A1 are long chain fatty acids (C_12 _to C_20_), which are exclusively hydroxylated at the subterminal positions (ω-1 to ω-3) (Boddupalli et al. 1990). Furthermore, this enzyme exhibits the highest catalytic activity ever detected among P450 monooxygenase (Boddupalli et al. 1990). Engineered CYP102A1 mutants derived by directed evolution and rational design could oxidize several non-natural substrates, including pharmaceuticals, short-chain hydrocarbons, and environmental chemicals (Yun et al. 2007; Stjernschantz et al. 2008; Seifert et al. 2009). The potential of engineered CYP102A1 for biotechnological applications has been recognized (Bernhardt 2006). Recently, it was reported that CYP102A1 can be developed as a potentially versatile biocatalyst for the generation of human P450 drug metabolites (Yun et al. 2007; Kim et al. 2009, 2010; Park et al. 2010; Sawayama et al. 2009; Whitehouse et al. 2009; Kim et al. 2011). Human P450 enzymes are responsible for the metabolism of about 75% of drugs used clinically (Williams et al. 2004; Guengerich 2003). Human drug metabolites are very useful in evaluating a drug's efficacy, toxicity, and pharmacokinetics (Johnson et al. 2004; Atrakchi 2009; Leclercq et al. 2009). They can also be used as starting materials for drug candidates.

By using a systematic screening strategy, we found a number of natural variants of CYP102A1. Although there were no apparent variations in hydroxylation activity towards myristic acid (C_14_) and palmitic acid (C_16_), the oxidation rates of lauric acid (C_12_) by the variants varied in the range of >25-fold. Some of the natural variants showed catalytic promiscuity towards non-natural substrates, particularly human P450 drug substrates. This study shows that diverse mutations are present in the gene of CYP102A1. Several specific residues for frequent mutations were found and the mutational frequency of reductase domains was much higher than that of heme domains.

## Materials and methods

### Materials

Isopropyl-β-D-thiogalactopyranoside (IPTG), glucose-6-phosphate, glucose-6-phosphate dehydrogenase, δ-aminolevulinic acid (δ-ALA), reduced β-nicotinamide adenine dinucleotide phosphate (NADPH), fatty acids, *N,O*-bis(trimethylsilyl)trifluoroacetamide (BSTFA), ferricyanide, phenacetin, acetaminophen, chlorzoxazone, coumarin, 7-ethoxycoumarin, and cytochrome *c *were obtained from Sigma-Aldrich (St. Louis, MO).

### Bacterial strains

Strains of *B. megaterium *used in this study were obtained from Korean Culture Center of Microorganisms (KCCM), Korean Collection for Type Cultures (KCTC), American Type Microbiology (ATCC), and the Institute of Fermentation, Osaka (IFO) (Table 1).

**Table 1:** *Bacillus megaterium *strains used in this study, and GenBank accession numbers for CYP102A1 variants, 16S rRNA, and ITS sequences between 16S-23S sequences^*a*^

		Accession Number		
		
Strain	Variant Name*^b^*	Genomic DNA	16S rRNA	16S-23S intergenic
KCCM 11745	102A1.1	(J04832)*^c^*	FJ917385	FJ969781
IFO 12108	102A1.1	(J04832)*^c^*	FJ969756	FJ969774
ATCC 14581	102A1.1	(J04832)*^c^*	FJ969751	FJ969767
KCCM 41415	102A1.1	(J04832)*^c^*	FJ969762	FJ969792
KCTC 3712	102A1.2	FJ899078	FJ969764	FJ969795
KCCM 12503	102A1.3	FJ899082	FJ969761	FJ969787
ATCC 15451	102A1.4	FJ899085	FJ969753	FJ969768
ATCC 10778	102A1.5	FJ899078	FJ969746	FJ969765
KCCM 11938	102A1.5	FJ899078	FJ969760	FJ969786
KCCM 11761	102A1.5	FJ899078	FJ969757	FJ969783
KCCM 11776	102A1.6	FJ899081	FJ969758	FJ969784
KCCM 11934	102A1.6	FJ899081	FJ969759	FJ969785
ATCC 14945	102A1.7	FJ899084	FJ969749	FJ969766
ATCC 21916	102A1.8	FJ899092	FJ969755	FJ969772
KCTC 2194	102A1.8	FJ859036	FJ969763	FJ969794
ATCC 19213	102A1.9	FJ899091	FJ969754	FJ969769
ATCC 12872	QM B1551*^d^*	-*^e^*	-*^e^*	-*^e^*

*^a^*GenBank accession numbers (except J04832) were assigned to nucleotide sequences determined in this study. The corresponding CYP102A1 variant gene for each strain is listed.

**Figure 1:**
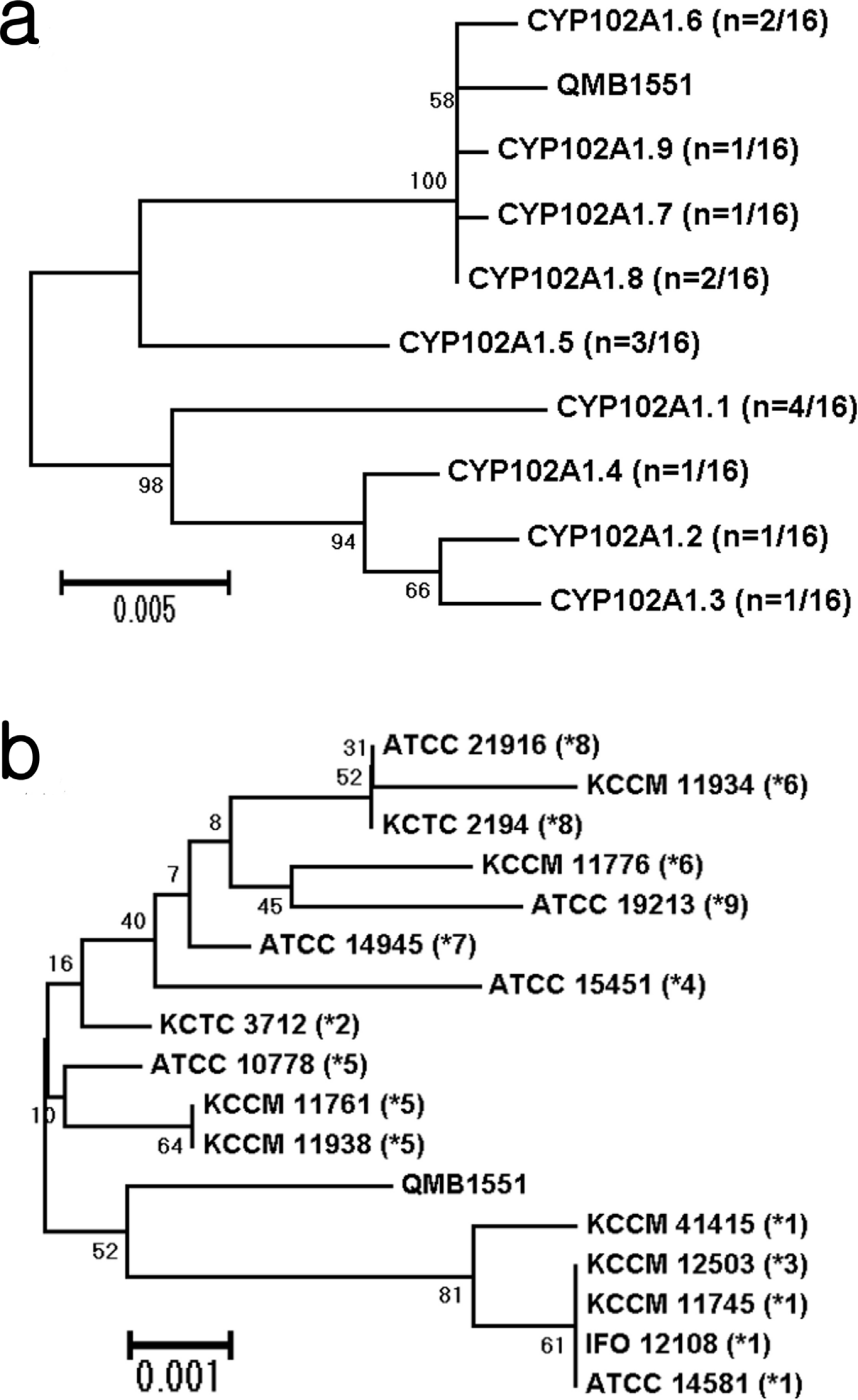
**Summarized phylogeny of CYP102A1 natural variants and intergenic sequence (ITS) alleles from *B. megaterium *strains**. (a) Phylogenetic analyses of CYP102A1 variants are based on the amino acid substitutions (Table 2 and Fig. S1) and silent mutations are excluded. Relative abundances are shown in parentheses. (b) Phylogenetic analyses of *B. megaterium *strains, which express CYP102A1, were based on the ITS gene sequences. The CYP102A1 variant expressed by each strain is shown as a number with an asterisk in parentheses. Numbers on tree branches show the percent bootstrap support for all branches important for interpretation. Nodes with bootstrap values of 1,000 resamplings (expressed by percentages) are indicated and the bar scales represent the substitution of amino acids (a) or nucleotides (b) per site.

*^b^*The CYP102A1 variants were named based on the amino acid similarity (Fig. 1a and Table 2).

*^c^*Previously known as the nucleotide sequence of P450 BM3 (CYP102A1) from *B. megaterium *(Ruettinger et al. 1989).

*^d^*Genetic Information regarding the CYP102A1 variant of *B. megaterium *QM B1551 (ATCC 12872) was obtained from the Whole Genome Sequencing of *B. megaterium *http://www.bios.niu.edu/b_megaterium/ and the variant was designated as QM B1551. We only used its genetic information to compare to those of other variants and did not study its biochemical and physical properties.

*^e^*Genetic information of *B. megaterium *QM B1551 (ATCC 12872) regarding its CYP102A1 variant, 16S rRNA, and ITS was obtained from the Whole Genome Sequencing of *B. megaterium *http://www.bios.niu.edu/b_megaterium/. Accession numbers were not provided.

### PCR and cloning of CYP102A1 natural variants

For DNA preparations, cells were grown in nutrient broth. After overnight growth at 37°C, the cells were centrifuged, washed, lysed, and enzymatically treated to remove RNA and protein. The DNA preparation was then treated with phenol-chloroform (50:50) and ethanol-precipitated. The purity was evaluated by measuring UV absorbance. The variant genes from *B. megaterium *were amplified by polymerase chain reaction (PCR) using oligonucleotide primers and *B. megaterium *chromosomal DNA template. First, PCR was carried out in a 50 μl reaction mixture containing template plasmid, forward primer BamHI-F (5'- AGCGGATCCATGACAATTAAAGAAATGCCTC-3') and reverse primer SacI-R (5'-ATCGAGCTCGTAGTTTGTAT-3'), dNTPs, and pfu polymerase. The PCR was carried out for 30 cycles consisting of 45 s of denaturation at 94°C, 45 s of annealing at 52°C, and 90 s of extension at 72°C. Next, PCR was carried out in a similar way by use of forward primer SacI-F (5'-ATACAAACTACGAGCTCGAT-3') and reverse primer XhoI-R (5'-ATCCTCGAGTTACCCAGCCCACACGTC-3'). The PCR product was digested with BamHI and SacI, and ligated into the pCW ori expression vector that had previously digested with the same restriction enzymes (Farinas et al. 2001). The amplified genes were subsequently cloned into the pCWBM3 BamHI/SacI vector at the BamHI/SacI restriction sites.

Because PCR amplification could lead to the introduction of random mutations and cloning of PCR products can fortuitously select the mutated sequences, all genes of CYP102A1 variants were PCR amplified a second time from genomic DNA and the sequences were directly determined without prior cloning. Exactly the same variations as those shown in Table 1 were again found, indicating that they were not artificially introduced during the PCR amplification.

### Sequencing and phylogenetic analysis of 16S rRNA and ITS between 16s and 23s rRNA

The amplification of partial 16S rRNA genes was carried out using the primers 9F (5'-GAGTTTGATCCTGGCTCAG-3') and 1512R (5'-ACGGCTACCTTGTTACGACTT-3') (Ni et al. 2008). The amplification reaction (25 μl) contained 50 ng DNA, 0.50 μM of each primer, 250 μM dNTPs, 1.5 mM MgCl_2_, and 1.25 U pfu polymerase in the buffer supplied by the manufacturer. The PCR was carried out for initial denaturation at 95°C for 5 min, followed by 30 cycles consisting of 95°C for 45 s, 55°C for 45 s, and 72°C for 90 s and final extension at 72°C for 10 min. Amplification products (10 μl) were electrophoresed in a 2% agarose gel and visualized under UV light after staining with ethidium bromide. Direct sequencing of the PCR products was performed with an ABI BigDye terminator v3.1 sequencing Ready Reaction kit.

One ITS region was amplified with primers 16S-F (5'- AAGTCGGTGGAGTAACCGT-3') and 23S-R (5'- TGTTAGTCCCGTCCTTCAT-3'). PCR reactions (25 μl) contained 50 ng DNA, 0.5 μM of each primer, 250 μM dNTPs, and 2.5 U Taq DNA polymerase in the buffer supplied by the manufacturer. The PCR was carried out for initial denaturation at 95°C for 15 min, followed by 35 cycles consisting of 95°C for 20 s, 52°C for 30 s, and 72°C for 60 s and final extension at 72°C for 3 min.

All sequencing procedures were repeated at least twice for each strain. The 16S rRNA gene sequences and the 16S-23S rRNA intergenic spacers were compared to sequences in the GenBank database using BLAST (Altschul et al. 1990). The sequences were aligned by using the CLUSTAL W program (Thompson et al. 1997).

### Expression and purification of CYP102A1 natural variants

Plasmids were transformed into *E. coli *DH5αF'-IQ cell. Overnight cultures (20 ml) grown in Luria-Bertani broth with ampicillin (100 μg/ml) selection at 37°C were used to inoculate a 250 ml culture of Terrific broth containing 100 μg/ml ampicillin, 1.0 mM thiamine, trace elements, 50 μM FeCl_3_, 1.0 mM MgCl_2_, and 2.5 mM (NH_4_)_2_SO. Cells were grown at 37°C and 250 rpm to an OD_600 _of between 0.6-0.8. Protein expression was induced by adding 1.0 mM IPTG and 1.5 mM δ-ALA, and cultures were grown at 28°C and 200 rpm for 50 h. The cells were harvested by centrifugation (15 min, 5,000 *g*, 4°C). The cell pellet was resuspended in TES buffer [100 mM Tris-HCl (pH 7.6), 500 mM sucrose, 0.5 mM EDTA] and lysed by sonication (Sonicator, Heat Systems - Ultrasonic, Inc.). After the lysate was centrifuged at 100,000 *g *(90 min, 4°C), the soluble cytosolic fraction was collected and used for the activity assay. The cytosolic fraction was dialyzed against 50 mM potassium phosphate buffer (pH 7.4) and stored at -80°C until use. The P450 concentration was determined by Fe^2+^-CO *versus *Fe^2+ ^difference spectra (Omura and Sato 1964).

### Binding affinity of fatty acids to CYP102A1 variants

To determine dissociation constants (*K*_d _values) of fatty acids to the CYP102A1 variants, spectral binding titration was performed for enzymes with saturated fatty acids (lauric acid, myristic acid, and palmitic acid). The *K*_d _values of substrates to the CYP102A1 variants were determined (at 23°C) by titrating 2.0 μM enzyme with the ligand, in a total volume of 1.0 ml of 100 mM potassium phosphate buffer (pH 7.4). The ligands were dissolved in dimethylsulfoxide and final dimethylsulfoxide concentrations were <1% (v/v). Absorbance increases at 390 nm and decreases at 420 nm as the substrate concentration increases (Lentz et al. 2001). The absorption difference between 390 nm and 420 nm was plotted against the substrate concentration (up to 1.0 mM) (Kim et al. 2008a, b). The *K*_d _values were determined from plots of induced absorption changes *versu*s ligand concentration. The data were fitted using a standard hyperbolic function or (where the *K*_d _value was within 5-fold of the P450 concentration) a quadratic function for tight-binding ligands, as described elsewhere (Girvan et al., 2010).

### Assay of fatty acid hydroxylation by natural variants and distribution of hydroxylated products

Metabolites were generated by incubation of 1.0 mM fatty acids and P450 enzyme (100 pmol) in a 1.0 ml volume of 100 mM potassium phosphate (pH 7.4) for 20 min at 37°C (Gustafsson et al. 2004). An aliquot of a NADPH-generating system was used to initiate reactions; final concentrations were 10 mM glucose 6-phosphate, 0.5 mM NADP^+^, and 1 IU/ml yeast glucose 6-phosphate dehydrogenase. The reactions were terminated with a 2-fold excess of ice-cold dichloromethane. After centrifugation of the reaction mixture, the organic solvent was removed under a gentle stream of nitrogen and the residue was dissolved in BSTFA (50 μl) containing trimethylchorosilane (1%, v/v). The solution was transferred to a glass vial and incubated at 75°C for 20 min to yield trimethylsilylated products. To determine the regioselectivity of hydroxylated products of fatty acids at the ω-1, ω-2, and ω-3 positions, GC/MS analysis was carried out on a Shimadzu QP2010 (column length, 30 m; internal diameter, 0.25 mm; film thickness, 0.1 μm), with electron-impact ionization. The GC oven temperature was programmed for 1 min at 70°C followed by an increase to 170°C at 25°C/min, to 200°C at 5°C/min, and to 280°C at 20°C/min. The oven was finally held at 280°C for 5 min. The MS source and interface were maintained at 250 and 280°C, respectively, and a solvent delay of 4 min was used. The mass spectra were collected using electron ionization at 70 eV. The products were identified by their characteristic mass fragmentation patterns (Lentz et al. 2001). Turnover numbers of the hydroxylation of fatty acids (lauric acid, myristic acid, palmitic acid) by the variants of CYP102A1 were determined by a GC-FID detector (Shimadzu GC2010 with FID detector). Essentially the same procedure was used for the regioselectivity of the hydroxylated products of fatty acid oxidation. The distribution of products was based on the relative peak area of the chromatogram of GC using hydroxylated products at ω position as standards.

### NADPH oxidation activities supported by natural variants

Reaction mixtures contained 1.0 mM fatty acid and P450 enzyme (25 nM) in a 1 ml volume of 100 mM potassium phosphate (pH 7.4). Initial rates of fatty acid-induced NADPH oxidation were measured by monitoring the absorption change at 340 nm (*ε*_340 _= 6,220 M^-1^cm^-1^) after NADPH was added at a concentration of 200 μM. Rates of change in *A*_340 _absorbance were converted into activity units (moles of NADPH oxidized per minute per mole of enzyme) (Noble et al. 1999).

### Enzymatic activities of reductase domains of natural variants

For the reductase assay, two different types of reductase substrates were used. One was a chemical substrate, ferricyanide, and the other was cytochrome *c*, which is a protein substrate, as described previously (Gustafsson et al. 2004). Assays for reductase domain-dependent electron transfer to exogenous electron acceptors (ferricyanide or cytochrome *c*) were also performed at 37°C in potassium phosphate (pH 7.4), with 2.5 nM enzyme, 200 μM NADPH, and electron acceptors (500 μM ferricyanide; 100 μM cytochrome *c*). Ferricyanide reduction was measured at 420 nm (*ε*_420 _= 1.02 mM^-1^cm^-1 ^for the ferricyanide reduction product) and cytochrome *c *reduction was measured at 550 nm (*ε*_550 _= 21.0 mM^-1^cm^-1 ^for the reduced cytochrome *c*).

### Thermal stability

To analyze enzyme stability, enzymes (2.0 μM) were incubated at different temperatures between 25 and 70°C for 20 min with subsequent cooling to 4°C in a PCR thermocycler (Eiben et al. 2007). The stability of the heme domain was calculated from heat-inactivation curves of CO-binding difference spectra (Omura and Sato 1964). The stability of the reductase domain was calculated from the reduction of ferricyanide catalyzed by reductase activity, as described above.

### Catalytic activity assays towards human P450 substrates

Purified natural variants of CYP102A1 were characterized for human P450 enzyme activities using specific substrates as summarized elsewhere (Yun et al. 2006): phenacetin *O*-deethylation for human P450 1A2; 7-ethoxycoumarin (7-EC) *O*-deethylation for human P450s 1A2, 2A6, and 2E1; 7-ethoxy-4-trifluoromethylcoumarin (7-EFC) *O*-deethylation for P450s 1A2 and 2B6; chlorzoxazone 6β-hydroxylation for P450 2E1; coumarin 7-hydroxylation for P450 2A6.

### Sequence analysis

DNA sequences of CYP102A1 variants, 16S rRNA sequences, and the ITS alleles between 16S and 23S rRNA genes obtained in this study were deposited in GenBank. The accession numbers are provided at Table 1. Genetic information of *B. megaterium *QM B1551 (ATCC 12872) regarding the CYP102A1 variant, 16S rRNA, and ITS was obtained from the homepage of Whole Genome Sequencing of *B. megaterium *http://www.bios.niu.edu/b_megaterium/.

The sequences were aligned using the MEGA 3.1 program (Molecular Evolutionary Genetic Analysis) (http://www.megasoftware.net/mega_dos.html). The size of CYP102A1 variants was 1,049 amino acids (Additional file 1). ITS (338 nucleotides) between 16S and 23S rRNA genes of *B. megaterium *was analyzed in this study. Phylogenetic trees were conducted by the neighbor-joining method using the MEGA 3.1 program. Bootstrap analysis of the neighbor-joining data, using 1,000 resamplings, was carried out to evaluate the validity and reliability of the tree topology.

### Nucleotide sequence accession numbers

The nucleotide sequences determined in this study have been deposited in the GenBank database (Table 1): FJ859036, FJ899078, FJ899080 to FJ899082, FJ899084, FJ899085, FJ899091, and FJ899092 for CYP102A1 variants; FJ917385, FJ969746, FJ969749, FJ969751, and FJ969753 to FJ969764 for 16S rRNA genes of *B. megaterium*; FJ969765 to FJ969769, FJ969772, FJ969774, FJ969781, FJ969783 to FJ969787, FJ969792, FJ969794, FJ969795 for ITS of 16S-23S rRNA genes of *B. megaterium*.

## Results

### Natural variants of CYP102A1 within a species of *B. megaterium*

Among 16 different strains of *B. megaterium*, 12 strains have natural genetic variants of CYP102A1 (Table 1). As some of them shared exactly the same DNA sequences, there were ultimately nine different types of CYP102A1 natural variants (Figure 1a, Table 1 and 2), including four previously reported variants (CYP102A1.1) (Ruettinger et al. 1989). Amino acid sequences of the CYP102A1 variants showed more than 96% identity with CYP102A1.1 (Table 2 and Additional file 1). The amino acid differences among the variants included 20 residues (CYP102A1.3, 20/1049, 1.9%) to 33 residues (CYP102A1.7, CYP102A1.8, CYP102A1.9; 33/1049, 3.1%) among a total of 1,049 amino acids (Table 2). Phylogenetic analyses of the amino acid sequences of CYP102A1 variants showed that three variants are closely related to CYP102A1.1 and five variants are distinct from it (Figure 1a). Among the total 55 mutated amino acid residues, those located in the reductase domains (residues 474-1049) (45 of 55, 82%) occurred at a much higher frequency than in heme domain (residues 1-473) (10 of 55, 18%) (Table 2). Interestingly, no substitutions in the amino acid residues of the active site or substrate channel (Ravichandran et al. 1993; Li and Poulos 1997) were seen among the 55 substitutions.

**Table 2:** Sequence variations of CYP102A1 variants*^a^*

	CYP102A1 Variants										
	**Mutated Amino acid**	**Change of Nucleotide**	*2	*3	*4	*5	*6	*7	*8	*9	**QMB1551**

	T2P	4A > C									+

**Heme domain**	V27I	79G > A	+		+		+	+	+	+	+
	
	A29T	85G > A	+		+		+	+	+	+	+
	
	V128I	382G > A	+		+	+	+	+	+	+	+
	
	A136T	406G > A	+		+		+	+	+	+	+
	
	E208D	624A > C				+					
	
	A222T	664G > A									+
	
	A296T	886G > A	+		+						
	
	D370E	1110C > A	+		+						
	
	K453Q	1357A > C				+	+	+	+	+	+
	
	T464R	1392T > A				+	+	+	+	+	+
	
	V471E	1413A > G				+	+	+	+	+	+

**Reductase domain**	K474T	1422G > C				+	+	+	+	+	+
	
	A475V	1424C > T	+	+	+	+	+	+	+	+	+
	
	Q513R	1539G > A						+			
	
	R526P	1578C > T					+				
	
	Q547E	1639C > G					+	+	+	+	+
	
	E559D	1677A > C	+	+	+						
	
	L590F	1794C > A								+	
	
	A591S	1771G > T				+					
	
	D600E	1800C > A				+	+	+	+	+	+
	
	V625L	1873G > T				+	+	+	+	+	+
	
	D632N	1894G > A				+					
	
	D638E	1914T > A					+	+	+	+	+
	
	K640A	1920A > T				+	+	+	+	+	+
	
	A652S	1954G > T									+
	
	G661R	1981G > C					+	+	+	+	+
	
	T665A	1993A > G	+	+	+	+	+	+	+	+	+
	
	Q675K	2023C > A					+	+	+	+	+
	
	P676L	2027C > T	+	+							
	
	A679E	2036C > A	+	+	+						
	
	E688A	2063A > C	+	+	+						
	
	T716A	2146A > G					+	+	+	+	+
	
	A717T	2149G > A				+	+	+	+	+	+
	
	A742G	2225C > G	+	+	+	+	+	+	+	+	+
	
	A783V	2348C > T					+	+	+	+	+
	
	A796T	2386G > A				+					
	
	K814E	2440A > G	+	+	+	+	+	+	+	+	+
	
	I825M	2474A > G				+	+	+	+	+	+
	
	R826S	2476C > A	+	+							
	
	R837H	2510G > A	+	+							
	
	E871N	2613G > T	+	+	+		+	+	+	+	+
	
	I882V	2644A > G	+	+	+	+	+	+	+	+	+
	
	E888G	2663A > G	+	+	+	+	+	+	+	+	+
	
	D894G	2681A > G					+	+	+	+	+
	
	P895S	2683C > T	+	+	+						
	
	G913S	2739C > T			+						
	
	E948K	2842G > A					+	+	+	+	+
	
	S955N	2864G > A	+	+	+	+	+	+	+	+	+
	
	M968V	2904G > A	+	+	+	+	+	+	+	+	+
	
	Q971E	2911C > G					+				
	
	M980V	2938A > G				+					
	
	Q982R	2945A > G	+	+							
	
	A1009D	3026C > A	+	+	+	+	+	+	+	+	+
	
	D1020E	3060C > A				+	+	+	+	+	+
	
	H1022Y	3066C > T	+	+	+						
	
	Q1023K	3067C > G				+					
	
	Q1023E	3067C > A	+	+	+						
	
	G1040S	3118G > A				+					

*^a^*Variations of amino acids and nucleotides in CYP102A1 variants (*2~*9) relative to CYP102A1.1 (P450 BM3) (*1) are shown by a (+) mark. Information regarding the CYP102A1 variant (designated as QMB1551) of *B. megaterium *QM B1551 (ATCC 12872) was obtained from the Whole Genome Sequencing of *B. megaterium *http://www.bios.niu.edu/b_megaterium/. We only used its genetic information to compare to those of other variants. Blanks mean no change of amino acids or nucleotides.

### Phylogenic analysis of bacterial strains and natural variants

**Figure 2:**
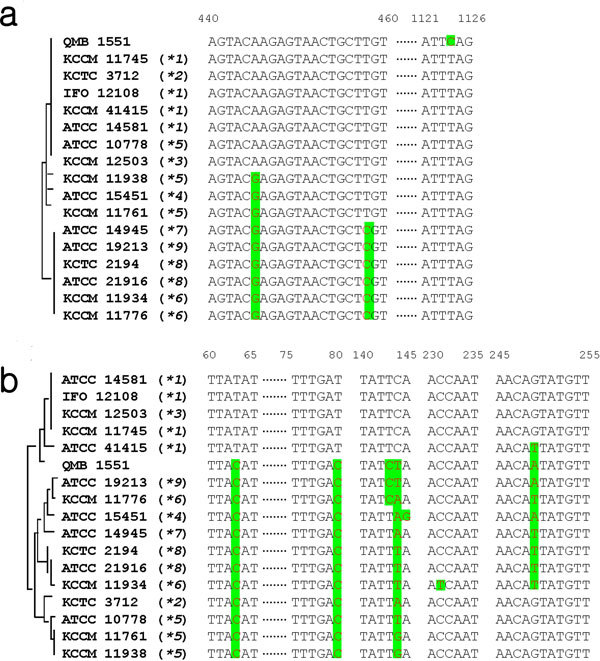
**Comparison of distinct regions of 16S rRNA gene sequences and ITS from *B. megaterium***. Two and seven nucleotides were variable among 1,394 and 338 nucleotides, respectively, of 16S rRNA (a) and ITS (b) genes of *B. megaterium *strains.

The 16S rRNA gene has been the molecular standard in studying evolutionary relationships among bacteria (Woese et al. 1990). Although DNA sequences of the 16S rRNA genes of 16 *B. megaterium *strains are well conserved (2 nucleotides are variable among a total of 1,394 nucleotides, 99.9% identity) (Figure 2a), the intergenic sequence (ITS_ alleles between 16S and 23S rRNA genes, which reflect the evolution of the bacterial strains (Gürtler 1999), showed 7 nucleotide variations among a total of 338 nucleotides (98.8% identity) (Figure 2b). Interestingly, the phylogenetic tree of ITS alleles was quite different from that of CYP102A1 natural variants. RNA analyses showed that the evolutionary profile of CYP102A1 variants is different from that of host strains (Figure 1).

### Biochemical characterization of the natural variants

**Figure 3:**
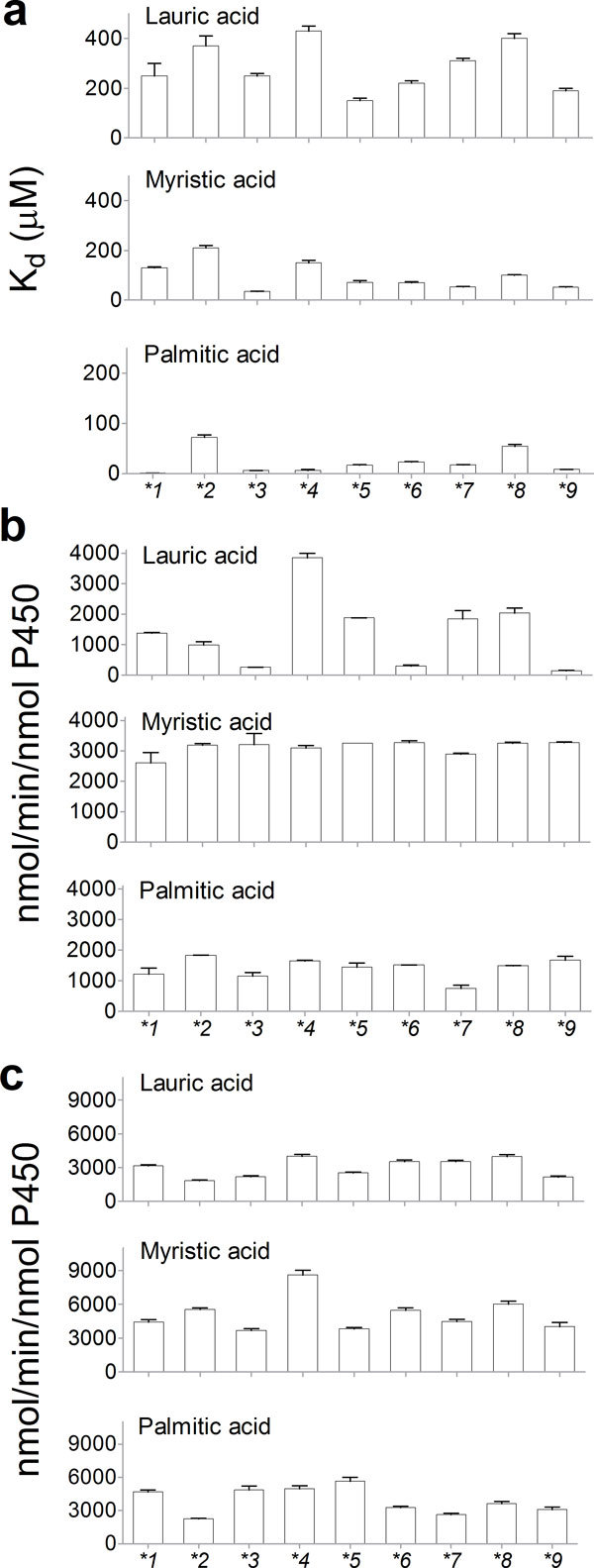
**Biochemical properties of natural variants**. (a) Dissociation constants (*K*_d _values) of substrates (lauric acid, myristic acid, and palmitic acid) to CYP102A1 natural variants. (b) Turnover numbers of the hydroxylation of fatty acids (lauric acid, myristic acid, palmitic acid) by the variants of CYP102A1. (c) Rates of fatty acid-dependent NADPH oxidation by the variants of CYP102A1.

The biochemical properties of the variants were examined. All CYP102A1 variants could bind to saturated fatty acids in the range of 12-16 carbons with a general preference for long fatty acids (Figure 3a). The affinity of the variants to the fatty acids was quite different from that of CYP102A1.1 in the range of >50-fold for palmitic acid. However, the variations were less than 5-fold for lauric acid and myristic acid.

Although there were no apparent variations in hydroxylation activity towards myristic acid (C_14_) and palmitic acid (C_16_), the oxidation rates of lauric acid (C_12_) by the variants varied in the range of >25-fold (Figure 3b). However, most of them did not show apparent changes in regioselectivity towards fatty acids (Additional file 2). For all fatty acids (C_12, _C_14_, C_16_) tested here, there were no apparent variations of regioselectivity among a set of CYP102A1 variants. CYP102A1 variants showed a preference for hydroxylation at the ω-1 position of lauric acid, and myristic acid, and at the ω-2 position for palmitic acid. Fatty acid-dependent NADPH oxidation rates by the variants were also determined in the presence of lauric, myristic, and palmitic acids (Kitazume et al. 2007) (Figure 3c). We could not find a direct correlation between NADPH oxidation and product formation of hydroxylated fatty acids.

The reductase activity towards ferricyanide was quite dependent on the type of CYP102A1 variant (Additional file 3). Variant CYP102A1.3 showed a 3-fold higher activity than that of CYP102A1.1. In the case of cytochrome *c*, variant CYP102A1.2 had the highest activity, which was 3-fold higher than that of CYP102A1.1. These variations seem to be related to the variations in amino acid sequence.

### Thermal stability of heme and reductase domains in the natural variants

**Figure 4:**
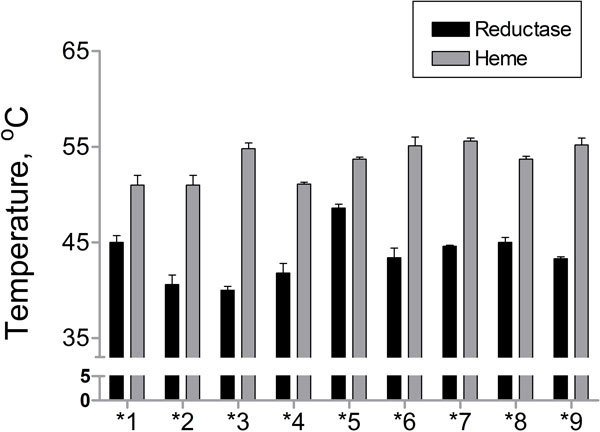
**Thermal stability for each domain of CYP102A1 variants**. Enzymes (2 μM) were incubated at different temperatures between 25 and 70°C for 20 min with subsequent cooling to 4°C in a PCR thermocycler. The stability of the heme domain was calculated from heat-inactivation curves of CO-binding difference spectra. The stability of the reductase domain was calculated from the reduction of ferricyanide catalyzed by reductase activity.

The thermal stability of the heme and reductase domains was examined. The *T*_50 _value of the CYP102A1.1 heme domain was 51°C and the variants showed similar *T*_50 _values in the range of 51-55°C (Figure 4). The *T*_50 _value of the CYP102A1.1 reductase domain was 45°C and the *T*_50 _values of the variants' reductase domains were in the range of 40-48°C. CYP102A1.5 (*T*_50_, 48°C) showed the highest thermal stability among CYP102A1 variants. The thermal stabilities of the reductase domains were much lower than those of the heme domains of the CYP102A1 variants.

### Catalytic promiscuity of the natural variants towards non-natural substrates

**Figure 5:**
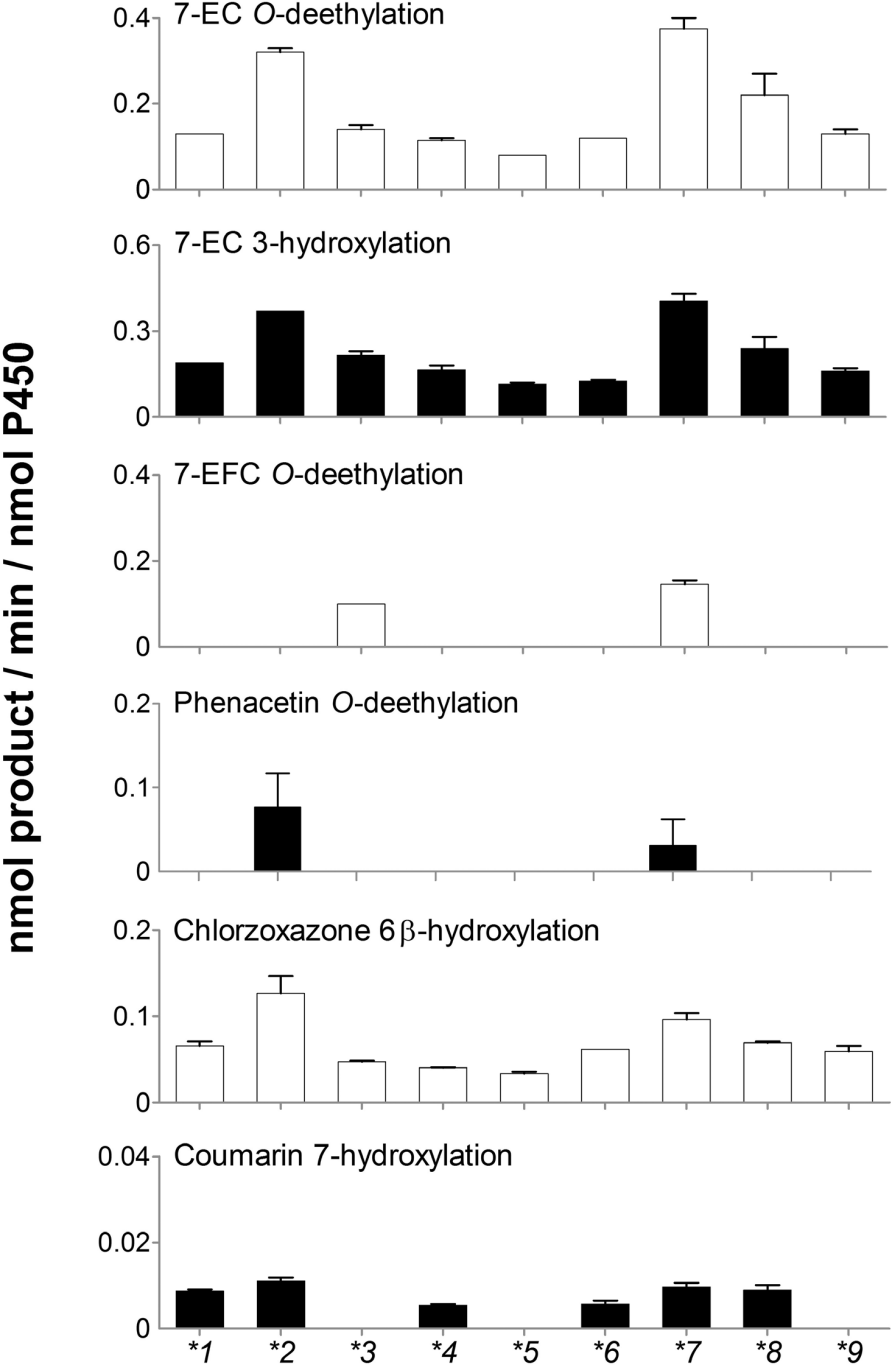
**Catalytic promiscuity of natural variants of CYP102A1 towards human P450 substrates**. Purified natural variants of CYP102A1 were characterized for human P450 enzyme activities using specific substrates: phenacetin *O*-deethylation for P450 1A2; 7-ethoxycoumarin (7-EC) *O*-deethylation for P450s 1A2, 2A6, and 2E1; 7-ethoxy-4-trifluoromethylcoumarin (7-EFC) *O*-deethylation for P450s 1A2 and 2B6; chlorzoxazone 6β-hydroxylation for P450 2E1; coumarin 7-hyroxylation for P450 2A6. Data are shown as the means ± SEM.

It is known that wild-type and several mutants of CYP102A1 could oxidize several human P450 substrates, including pharmaceuticals (Yun et al. 2007). We examined the catalytic promiscuity of the CYP102A1 variants towards non-natural substrates. They showed quite distinct catalytic activities towards typical human P450 substrates including drugs (Figure 5). CYP102A1.7 could oxidize all human P450 substrates tested here. Although the oxidation rates of the variants for all tested human P450 substrates were fairly low (< 0.4 min^-1^), we detected potential evidence for the evolvability of P450 catalytic activities. Low catalytic activity is an intrinsic property of human P450 enzymes (Guengerich 2005). This result indicates that the variants show catalytic promiscuity towards non-natural substrates.

## Discussion

The current study provides a glimpse into P450 diversity in bacteria. Extensive diversity of P450 genes has been found in bacteria, including a large set of strains of the genus *Bacillus *(Porwal et al. 2009). As we begin to survey the variants of bacterial P450 enzymes through a systematic approach with *B. megaterium *strains, there are exciting opportunities for studying the catalytic capabilities and the metabolic functions of the P450 monooxygenase systems. This work shows the presence of a number of P450 natural variants within a species of *B. megaterium*. Multiple amino acid substitutions (up to 4 among 528 amino acids of *Candida albicans*) in a fungal CYP51 (Kelly et al. 2005) and a large number of alleles in human P450 (Human Cytochrome P450 Allele Nomenclature Committee; http://www.cypalleles.ki.se/) and human NADPH-P450 reductase (Huang et al. 2008) genes were found. However, the diversity of a P450 gene within a species is much lower in these species than in *B. megaterium *CYP102A1.

Phylogenetic analysis suggests that CYP102A1 gene could have evolved more rapidly than the rRNA gene locus of the host strains under the selective pressures of their environments. For example, *B. megaterium *strains IFO 12108 (and KCCM 11745) and KCCM 12503 have exactly the same 16S rRNA genes and ITS, but they express different variants of CYP102A1.1 and CYP102A1.3, respectively (Figure 1b and 2). Given the diversification of ITS alleles that accompanies the strain evolution of *B. megaterium*, the distribution of CYP102A1 variants should uniquely define particular clades (Figure 1 and 2).

The reductase domains of CYP102A1 variants are more divergent than heme domains (Table 2 and Additional File 1). However, binding sites of heme, FMN, and FAD, which are essential cofactors for oxidation activities, are well conserved except for a few residues of the FAD binding site of CYP102A1. Substitutions of amino acids in reductase domains of CYP102A1 variants occurred at high frequency (7.8% of total amino acid residues). Mutations at the reductase domain may influence the monooxygenase activity of heme domain by controlling electron transfer process from reductase domain to heme domain. The changes in activity due to the mutations might give the organism a selective advantage for the evolutionary adaptation driven by different metabolic or environmental demands. In addition, the results of thermal stability (Figure 4) suggest that the higher mutation rate of the CYP102A1 reductase domain might affect the thermal stability of the reductase domains.

The occurrence of multiple amino acid substitutions appears to be common in CYP102A1 natural variants, although it is unclear as yet whether all identified mutations are important for substrate affinity, thermal stability, catalytic activities, and their promiscuity to non-natural substrates. It is found that wild-type CYP102A1 can catalyze the hydroxylation of chlorzoxazone, aniline and *p*-nitrophenol, as well as the *N*-dealkylation of propranolol and the dehydrogenation of nifedipine. These chemicals are typical substrates of human P450s 2E1, 2D6, 1A2 and 3A4, which are the main drug-metabolizing enzymes. The catalytic activities of P450 BM3 are either comparable or higher than those measured for the human enzymes towards these smaller and non-physiological substrates. These results suggested the possibility to obtain fine chemicals including human drug metabolites by using CYP102A1 (Yun et al., 2007) and references therein). It should also be noted that highly active mutants of CYP102A1.1 (P450 BM3), which were obtained by directed evolution using random mutagenesis, towards non-natural substrates such as short-chain hydrocarbons (Peters et al. 2003), drugs (van Vugt-Lussenburg et al. 2007), and xenobiotics (Whitehouse et al. 2008) contained mutations that are not located in the active site.

Substrate and catalytic promiscuities are believed to be hallmark characteristics of primitive enzymes, serving as evolutionary starting points from which greater specificity is acquired following application of selective pressures (Khersonsky et al. 2006). It was proposed that the evolution of a new function is driven by mutations that have little effect on the native function but large effects on the promiscuous functions that serve as the starting point (Aharoni et al. 2005). Here we propose an alternative view of P450 evolution by which bacterial P450 enzymes acquire a new catalytic activity through mutations besides the crucial catalytic residues of the substrate binding region, substrate channel, and active site. This hypothesis may also provide clues to explain how P450 enzymes show broad substrate specificity, a characteristic that is specific to the P450 enzymes (Guengerich 2001). Catalytic promiscuity of bacterial P450s, at least CYP102A1, seems to be intrinsic to P450s, although the mechanisms by which the mutations contribute to the new activity are difficult to rationalize.

Here we report the presence of diverse natural variants of CYP102A1 within a species of *B. megaterium*. Phylogenetic analyses suggest that the CYP102A1 gene evolves more rapidly than the rRNA gene locus. While key catalytic residues in the substrate channel and active site are retained, several specific residues for frequent mutation were found. Although there were no apparent variations in hydroxylation activity towards myristic acid (C_14_) and palmitic acid (C_16_), the hydroxylation rates of lauric acid (C_12_) by the variants varied in the range of >25-fold. Furthermore, catalytic activities of the variants are promiscuous towards non-natural substrates including human P450 substrates. These results suggest that bacterial P450 enzymes can acquire new catalytic activities through site-specific mutations distal to the active site. As these natural variants show similar activities as human P450 enzymes, they can be developed as industrial enzymes for cost-effective and scalable production of fine chemicals including drugs and their metabolites. Combined with rational design and directed evolution, the catalytic promiscuity of the self-sufficient CYP102A1 enzyme can be useful for extending their application in several fields of biotechnology.

## List of abbreviations

P450 or CYP: Cytochrome P450s; CYP102A1: P450 BM3; IPTG: isopropyl-β-D-thiogalactopyranoside; δ-ALA: δ-aminolevulinic acid; NADPH: reduced β-nicotinamide adenine dinucleotide phosphate; BSTFA: *N,O*-bis(trimethylsilyl)trifluoroacetamide; KCCM: Korean Culture Center of Microorganisms; KCTC: Korean Collection for Type Cultures; ATCC: American Type Microbiology; IFO: Institute of Fermentation, Osaka; PCR: polymerase chain reaction; ITS: intergenic sequence; *K*_d_: dissociation constants; 7-EC: 7-ethoxycoumarin; 7-EFC: 7-ethoxy-4-trifluoromethylcoumarin.

## Competing interests

The authors declare that they have no competing interests.

## Authors' contributions

JYK carried out the molecular genetic studies and the assay of fatty acid hydroxylation. SYK carried out the molecular genetic studies and the assay of fatty acid hydroxylation. DK participated in the sequence alignment and performed the statistical analysis. DHK carried out the molecular genetic studies and the assay of fatty acid hydroxylation. SMS carried out the molecular genetic studies and the assay of fatty acid hydroxylation. SHP carried out the assay of human drug substrate oxidation. KHK carried out the assay of human drug substrate oxidation. HCJ conceived of the study and participated in the design of the study. JGP participated in the design of the study. YHJ conceived of the study and participated in the design of the study. YTC participated in the design of the study. HZC participated in the design of the study. TA participated in the design of the study. CHY conceived of the study, participated in its design and coordination and wrote the manuscript. All authors read and approved the final manuscript.

## Authors' information

CHY received a Ph.D. from Korea Advanced Institute of Science and Technology (Korea) with a major in biochemistry at 1990. Under the supervision of Prof. Hyoungman Kim, his thesis work focused on the protein interactions with phospholipids. Thereafter, he pursued post-doctoral studies with Prof. F. Peter Guengerich at Vanderbilt University (Nashville, TN, USA), where he studied human P450 enzymes. During 1992-2004, he took a position as a Professor at Paichai University (Daejeon, Korea). In 2004 he joined Chonnam National University (Gwangju, Korea) as a Professor at the School of Biological Sciences and Technology. His current research interests include the use of bacterial P450 enzymes as novel biocatalysts and their use in the production of drug metabolites.

## Supplementary Material

Additional file 1 **Amino acid sequence alignment of CYP102A1 and its variants**. CYP102A1 variants are arranged in order corresponding to the molecular phylogeny (Figure 1a) as indicated by the simplified schematic to the left of the amino acid alignment. Secondary structures are shown below the CYP102A1 variant sequences: α-helices, red; β-sheets, blue. Binding sites of cofactors are shown: heme (yellow), FMN (dark blue), and FAD (gray). Click here for file

Additional file 2 **Distribution of hydroxylated products of fatty acids by CYP102A1 variants**. Regioselectivity of the hydroxylated products of fatty acids at positions ω-1, ω-2, and ω-3 was determined. Click here for file

Additional file 3 **Enzymatic activities of the reductase domains of CYP102A1 variants**. Assays for reductase domain-dependent electron transfer to exogenous electron acceptors (ferricyanide or cytochrome *c*) were performed. Click here for file
