# Supplementary material for: Characterization of diverse natural variants of CYP102A1 found within a species of Bacillus megaterium
Source: AMB Express. 2011 Mar 28;1:1. doi: 10.1186/2191-0855-1-1 (PMC3159907; doi:10.1186/2191-0855-1-1)
Supplement: Additional file 1 — Amino acid sequence alignment of CYP102A1 and its variants. CYP102A1 variants are arranged in order corresponding to the molecular phylogeny (Figure 1a) as indicated by the simplified schematic to the left of the amino acid alignment. Secondary structures are shown below the CYP102A1 variant sequences: α-helices, red; β-sheets, blue. Binding sites of cofactors are shown: heme (yellow), FMN (dark blue), and FAD (gray). [file 2191-0855-1-1-S1.PDF]

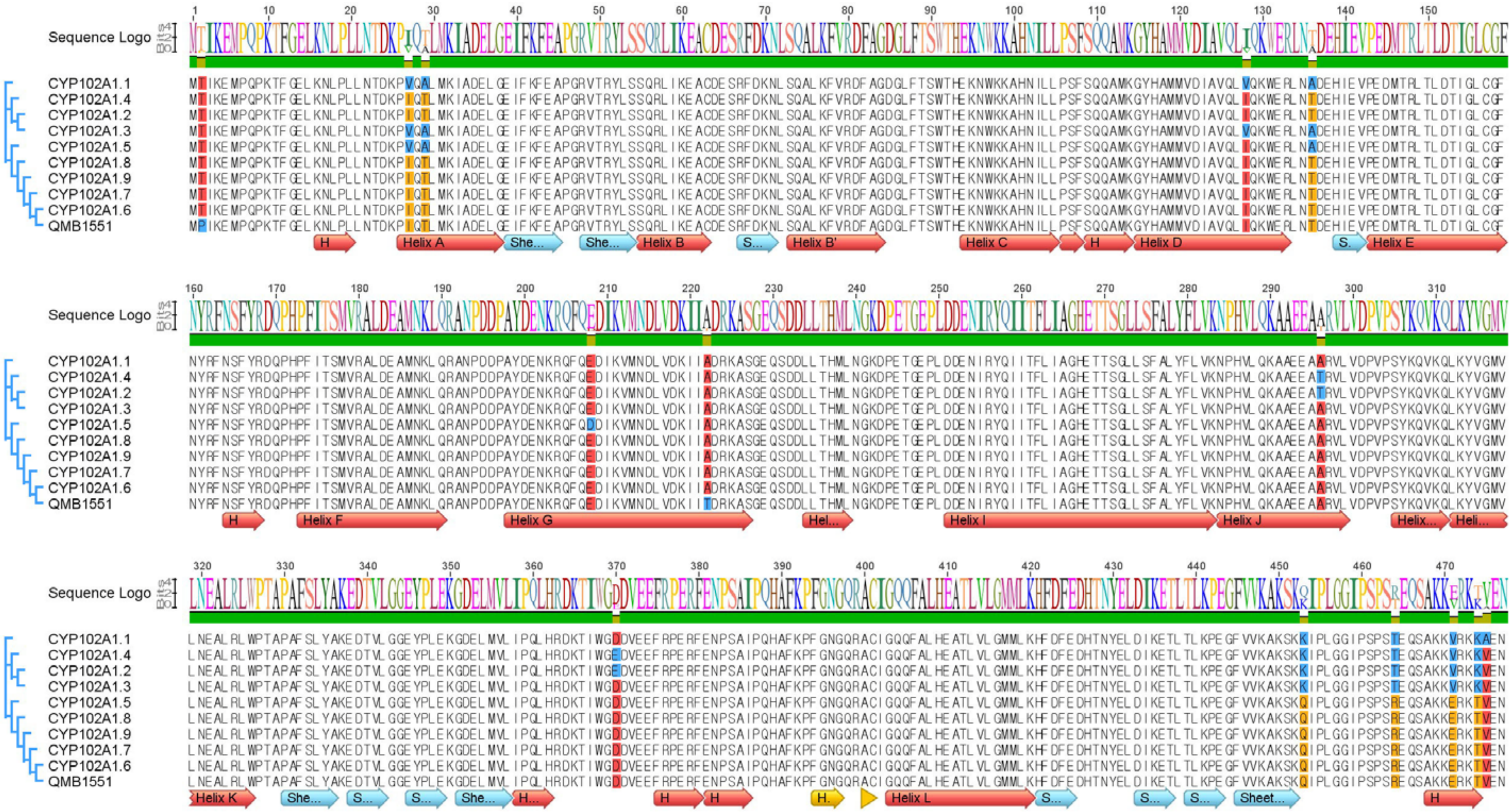
Sequence Logo
Bits
CYP102A1.1
CYP102A1.4
CYP102A1.2
CYP102A1.3
CYP102A1.5
CYP102A1.8
CYP102A1.9
CYP102A1.7
CYP102A1.6
QMB1551
Helix A
Helix B
Helix B'
Helix C
Helix D
Helix E
Helix F
Helix G
Helix I
Helix J
Helix K
Helix L
Sheet...

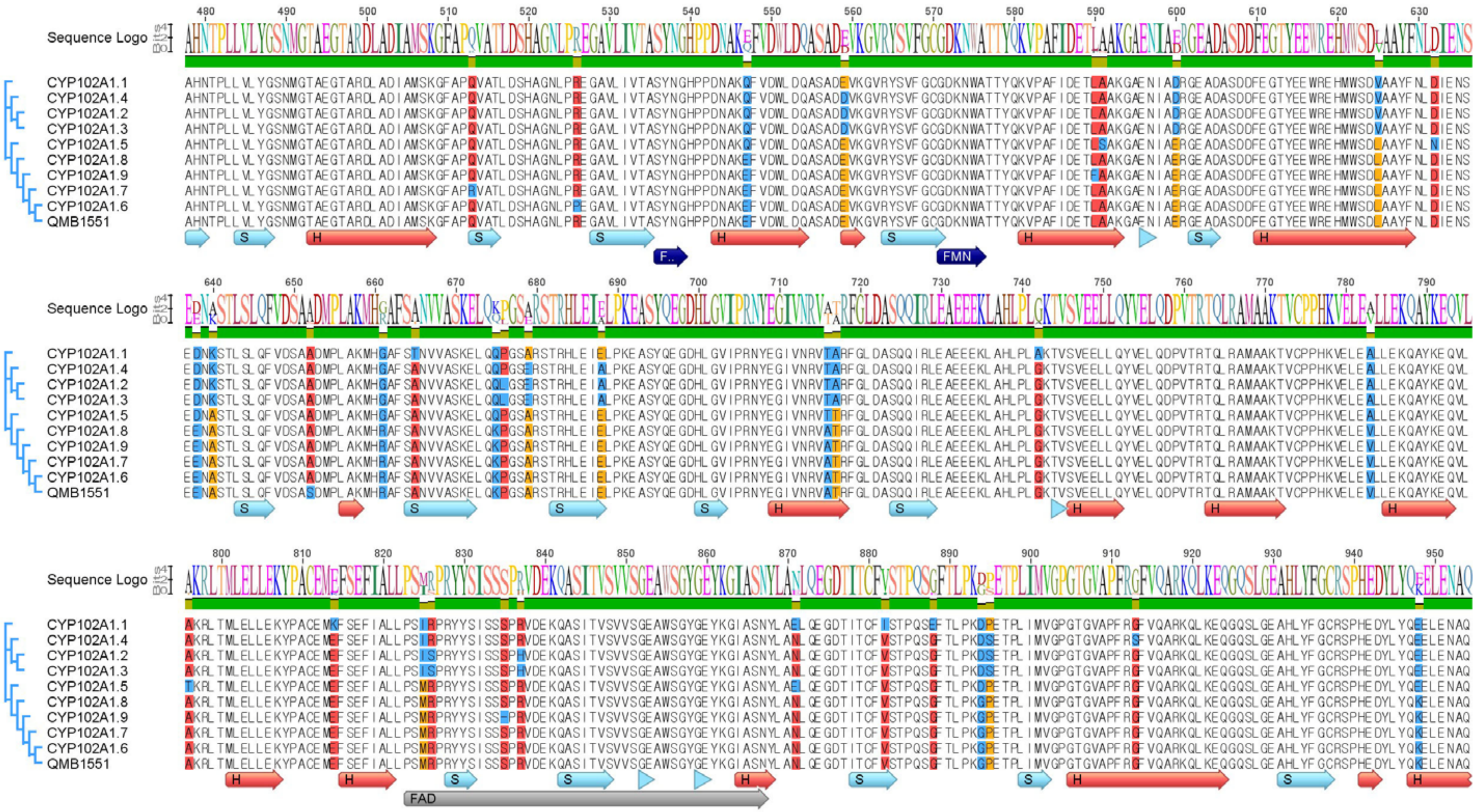

Sequence Logo
Bits
CYP102A1.1
CYP102A1.4
CYP102A1.2
CYP102A1.3
CYP102A1.5
CYP102A1.8
CYP102A1.9
CYP102A1.7
CYP102A1.6
QMB1551
FMN
FAD

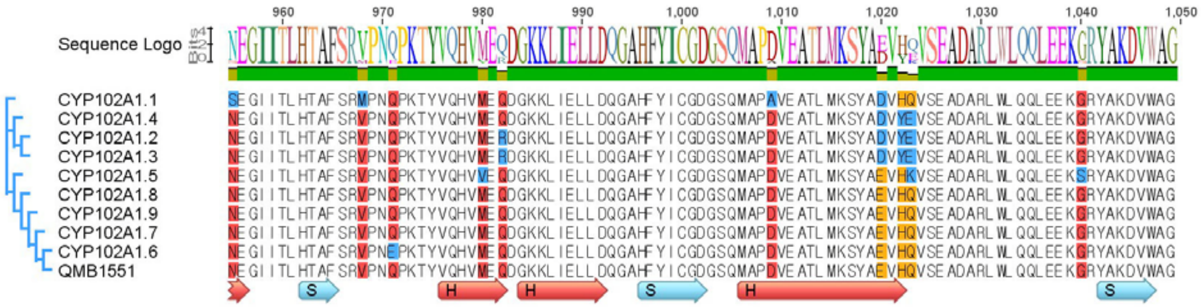

Additional file 1. Amino acid sequence alignment of CYP102A1 and its variants. CYP102A1 variants are arranged in order corresponding to the molecular phylogeny (Fig. 1a) as indicated by the simplified schematic to the left of the amino acid alignment. Secondary structures are shown below the CYP102A1 variant sequences: α-helices, red; β-sheets, blue. Binding sites of cofactors are shown: heme (yellow), FMN (dark blue), and FAD (gray).
