# Supplementary material for: Characterization of diverse natural variants of CYP102A1 found within a species of Bacillus megaterium
Source: AMB Express. 2011 Mar 28;1:1. doi: 10.1186/2191-0855-1-1 (PMC3159907; doi:10.1186/2191-0855-1-1)
Supplement: Additional file 2 — Distribution of hydroxylated products of fatty acids by CYP102A1 variants. Regioselectivity of the hydroxylated products of fatty acids at positions ω-1, ω-2, and ω-3 was determined. [file 2191-0855-1-1-S2.PDF]

**Additional File 2**

Distribution of hydroxylated products of fatty acids by CYP102A1 variants[a]

| | Lauric acid ($C_{12}$) | | | Myristic acid ($C_{14}$) | | | Palmitic acid ($C_{16}$) | | |
|---|---|---|---|---|---|---|---|---|---|
| Variants | ω-1 | ω-2 | ω-3 | ω-1 | ω-2 | ω-3 | ω-1 | ω-2 | ω-3 |
| CYP102A1.1 | 36 | 30 | 34 | 45 | 29 | 26 | 35 | 50 | 15 |
| CYP102A1.2 | 42 | 30 | 28 | 39 | 31 | 30 | 38 | 48 | 14 |
| CYP102A1.3 | 37 | 30 | 33 | 45 | 29 | 26 | 36 | 50 | 14 |
| CYP102A1.4 | 37 | 29 | 34 | 42 | 31 | 27 | 38 | 48 | 14 |
| CYP102A1.5 | 42 | 30 | 28 | 44 | 29 | 27 | 36 | 50 | 14 |
| CYP102A1.6 | 42 | 30 | 28 | 41 | 31 | 28 | 38 | 48 | 14 |
| CYP102A1.7 | 43 | 30 | 27 | 43 | 31 | 26 | 39 | 47 | 14 |
| CYP102A1.8 | 42 | 30 | 28 | 42 | 31 | 27 | 38 | 48 | 14 |
| CYP102A1.9 | 43 | 30 | 27 | 41 | 31 | 28 | 37 | 48 | 15 |

[a]Regioselectivity of the hydroxylated products of fatty acids at positions ω-1, ω-2, and ω-3 was determined. Values are the means of three independent experiments. All standard deviations were less than 10% of the average.
