# Supplementary material for: Characterization of diverse natural variants of CYP102A1 found within a species of Bacillus megaterium
Source: AMB Express. 2011 Mar 28;1:1. doi: 10.1186/2191-0855-1-1 (PMC3159907; doi:10.1186/2191-0855-1-1)
Supplement: Additional file 3 — Enzymatic activities of the reductase domains of CYP102A1 variants. Assays for reductase domain-dependent electron transfer to exogenous electron acceptors (ferricyanide or cytochrome c) were performed. [file 2191-0855-1-1-S3.PDF]

Additional file 3

Enzymatic activities of the reductase domains of CYP102A1 variants[a]

	k_{cat} (min^{-1})	
Variants	Ferricyanide	Cytochrome c
CYP102A1.1	27,300 ± 1,100	4,320 ± 500
CYP102A1.2	48,700 ± 2,200	13,000 ± 830
CYP102A1.3	95,500 ± 7,900	6,510 ± 740
CYP102A1.4	33,300 ± 1,600	3,410 ± 130
CYP102A1.5	18,700 ± 930	3,230 ± 460
CYP102A1.6	33,800 ± 1,200	9,490 ± 960
CYP102A1.7	36,500 ± 1,300	10,650 ± 900
CYP102A1.8	26,100 ± 1,100	5,190 ± 490
CYP102A1.9	18,700 ± 760	4,710 ± 270

[a]Values are means ± SEM of three independent experiments.
